# Diabetes in the older patient: heterogeneity requires individualisation of therapeutic strategies

**DOI:** 10.1007/s00125-018-4547-9

**Published:** 2018-02-07

**Authors:** Guntram Schernthaner, Marie Helene Schernthaner-Reiter

**Affiliations:** 1 Department of Medicine 1, Rudolfstiftung Hospital, Juchgasse 25, 1030 Vienna, Austria; 2 Clinical Division of Endocrinology and Metabolism, Department of Internal Medicine III, Medical University of Vienna, Währinger Gürtel, 18-20 1090 Vienna, Austria

**Keywords:** Age, Chronic kidney disease, Frailty, Glycaemic target, Hypoglycaemia, Older people, Review, Type 2 diabetes mellitus

## Abstract

Owing to the worldwide increase in life expectancy, the high incidence of diabetes in older individuals and the improved survival of people with diabetes, about one-third of all individuals with diabetes are now older than 65 years. Evidence is accumulating that type 2 diabetes is associated with cognitive impairment, dementia and frailty. Older people with diabetes have significantly more comorbidities, such as myocardial infarction, stroke, peripheral arterial disease and renal impairment, compared with those without diabetes. However, as a consequence of the increased use of multifactorial risk factor intervention, a considerable number of older individuals can now survive for many years without any vascular complications. Given the heterogeneity of older individuals with type 2 diabetes, an individualised approach is warranted, which must take into account the health status, presence or absence of complications, and life expectancy. In doing so, undertreatment of otherwise healthy older individuals and overtreatment of those who are frail may be avoided. Specifically, overtreatment of hyperglycaemia in older patients is potentially harmful; in particular, insulin and sulfonylureas should be avoided or, if necessary, used with caution. Instead, glucose-dependent drugs that do not induce hypoglycaemia are preferable since older patients with diabetes and impaired kidney function are especially vulnerable to this adverse event.

**Electronic supplementary material:**

The online version of this article (10.1007/s00125-018-4547-9) contains peer-reviewed but unedited supplementary material including a slide of the figure for download, which is available to authorised users.

## Introduction

In recent decades worldwide rates of diabetes have been on the rise, especially among older people. The global prevalence of diabetes is estimated to increase to 642 million by 2040 and the largest age-specific rise is predicted to be among people aged between 60 and 79 years [1]. Currently, approximately 20% of people between 70 and 79 years are thought to have diabetes [1]. The ageing of the population is thought to be one of the most important contributors to the prevalence of diabetes, since increasing age is a substantial risk factor for the development of this disease [1]. In the USA, more than one-third of the adult population with diabetes is currently aged 65 years or older, comprising almost 11 million people [2]. A very high prevalence of type 2 diabetes in older individuals is seen not only in the Western world, where economic standards are high, but also in developing countries, such as Brazil [3] and China [4]. In Brazil, almost 3 million of the 12 million people with diabetes are older than 65 years [3], whereas, in China, 35 million of the 92 million people with diabetes are older than 60 years and 20 million are older than 70 years [4].

In this article, we review the literature on diabetes in older people. We initially preselected about 500 papers in PubMed using the search terms ‘diabetes and age’, ‘ageing in diabetic patients’, ‘treatment of elderly patients’ and ‘co-morbidity of elderly patients’. Finally, about 100 references were used; RCTs and recent publications in high-ranking journals were preferred.

## Pathophysiology and types of diabetes in older people

The vast majority of older adults with diabetes have type 2 diabetes (>90%), owing to a combination of increased insulin resistance and impaired insulin secretion. Insulin resistance that is associated with advancing age is believed to be due to a combination of adiposity, sarcopenia (decreased muscle mass) and physical inactivity [5]. Impaired pancreatic beta cell adaptation to insulin resistance appears to be an important contributing factor to age-related glucose intolerance and risk of diabetes [6].

The identification of individuals with latent autoimmune diabetes of adults (LADA) is relevant for therapeutic decisions [7], since these individuals need insulin therapy much earlier than those with classical type 2 diabetes. Furthermore, since the longevity of people with childhood autoimmune diabetes has improved considerably over recent decades [8], an increasing proportion of older individuals with diabetes are those with classical type 1 diabetes.

## Comorbidities in older people with diabetes

The population of older people with type 2 diabetes consists of a spectrum of different disease severities between two extremes: those with long-standing type 2 diabetes since middle age and those with incident type 2 diabetes that only develops in older age. There are clear differences in the comorbidities and ease of glycaemic control in these two different type 2 diabetes categories, underlining that one-size-fits-all treatment guidelines are not appropriate in this age group.

### Vascular complications

If well treated for all cardiovascular risk factors from the time of diagnosis of diabetes, a considerable number of older people with diabetes remain free from severe vascular complications and can survive for many years [9]. By contrast, those who have not been well treated over an extended period can develop a range of macrovascular and microvascular complications. In the prospective GERODIAB observational study (*n* = 997 participants with type 2 diabetes; median age, 77 years old), the frequencies of all cardiovascular complications increased from 47% to 67% during the 5 year follow-up, including CHD, peripheral vascular disease and cerebrovascular disease [10]. Heart failure more than doubled during follow-up (9% to 20%) and was the strongest predictor of poor survival. Amputation and foot wounds were also strongly associated with poor survival [10].

### Cognitive impairment and dementia

Evidence is accumulating that type 2 diabetes is associated with cognitive impairment and dementia. Numerous epidemiological studies have demonstrated that people with type 2 diabetes have a significantly higher risk of developing Alzheimer’s disease [11, 12]. Dementia affects up to 16% of individuals with diabetes aged >65 and 24% aged >75 [13], and evidence shows that diabetes and dementia share a pathophysiological link [12]. Higher glucose levels were found to be associated with an increased risk of dementia in populations with and without diabetes [14], with this association being stronger in those with diabetes. Moreover, insulin resistance is also an important risk factor for cognitive impairment in older people with type 2 diabetes [15]. On the other hand, however, prospective studies have shown that severe hypoglycaemia is also a risk factor for cognitive impairment and dementia [16, 17]. Since cognitive dysfunction affects treatment adherence and diabetes self-management, the resulting poor glycaemic control and an increased rate of severe hypoglycaemia contribute to a vicious cycle. Overall, individuals with cognitive dysfunction have difficulty performing self-care (e.g. matching insulin dosage to carbohydrate intake or avoiding and treating hypoglycaemia), leading to a significantly reduced quality of life [18].

### Frailty

Frailty is a state of increased vulnerability to minor stressors, leading to difficulties in maintenance of homoeostasis, which increases the risk of adverse outcomes (disability, falls and death). When frailty occurs in older people with diabetes, sarcopenia or loss of muscle mass seems to be accelerated. In the Canadian Study of Health and Aging [19], the median life expectancy of frail older adults with diabetes was only 23 months. Recent studies suggest that crosstalk between insulin resistance, adipose tissue inflammation and skeletal muscle inflammation and dysfunction is involved in the development of sarcopenia and frailty [20]. Poor glycaemic control should be avoided, since in the Cardiovascular Health Study, an HbA_1c_ ≥ 63.9 mmol/mol (≥8.0%) (vs <36.6 mmol/mol [<5.5%]) was associated with a threefold increased risk of incident frailty and a three- to fivefold increased risk of lower extremity mobility limitations [21]. Frailty is also strongly associated with the presence of chronic kidney disease (CKD); it occurs in 21% of those with an eGFR <45 ml min^−1^ [1.73 m]^−2^ [22]. The appearance of frailty can change the natural history of type 2 diabetes, from a progressive to a regressive course with increased risk of hypoglycaemia [23]; declining body function associated with weight loss and malnutrition may lead to normoglycaemia and an increased risk of hypoglycaemia. In this situation it is important to reduce any hypoglycaemic medication or even withdraw it when necessary.

## Glycaemic control to reduce mortality rates in older people with type 2 diabetes

In the last 20 years, rates of all diabetes-associated vascular complications [24], as well as all-cause and cardiovascular mortality [25–28], have declined significantly in countries with high medical and economic standards, most likely because polypharmacy has reduced cardiovascular risk factors such as hypertension and dyslipidaemia. As a result of the considerably improved prognosis and longer survival of people with type 2 diabetes, in the future we will be confronted by a much higher proportion of older patients. The risk associated with diabetes and its comorbidities is well illustrated by two large studies of populations with very different socioeconomic backgrounds: a recent nationwide study from Sweden showing the 5 year follow-up of 450,000 older people with type 2 diabetes [9] and a prospective 12 year study from Mexico City that included >19,000 people with type 2 diabetes [29] (summarised in Table 1). In contrast to the Swedish study, where encouragingly the excess mortality owing to diabetes was low, a very different picture emerged in Mexico City, with a high excess mortality rate, worse glycaemic control and much lower rates of antihypertensive and lipid-lowering medication (Table 1). These findings highlight the importance of adequate glycaemic control in combination with good control of cardiovascular risk factors. Interestingly, the data from Mexico City showed a much higher excess mortality from renal disease than from cardiovascular disease (CVD) in study participants.

**Table 1:** Risk associated with diabetes and its comorbidities (Sweden vs Mexico City)

Variable	Sweden [9]		Mexico City [29]	
	Participants with diabetes	Control group	Participants with diabetes	Control group
*n*	435,369	2,117,483	19,068	126,978
Age (years)	65.8	65.5	59	51
Follow-up time (years)	4.6	4.8	12	NR
Diabetes duration (years)	6	–	9	–
HbA_1c_ (mmol/mol)	54.3	NR	74.9	37.7
HbA_1c_ (%)	7.1	NR	9.0	5.6
BP (mmHg)	140/79	NR	133/84	130/84
LDL-cholesterol, (mmol/l [mg/dl])	2.94 (113.5)	NR	NR	NR
Mortality (%)	17.7	14.5	19.9	4.6
Unadjusted excess mortality (%)^a^	3.2		15.3	
Cardiovascular mortality (%)	7.9	6.1	6.2	1.4
Unadjusted excess mortality (%)^a^	1.8		4.8	
Renal mortality (%)	NR	NR	5.4	0.3
Unadjusted excess mortality (%)^a^	–		5.1	
Mortality in diabetic individuals ≥75 years (%)	38.7	37.2	71.1	36.3
Unadjusted excess mortality (%)*	1.5		34.8	
Insulin use (%)	19.8	–	7.1	–
Oral glucose-lowering agents (%)	51.5	–	NR	–
Biguanide use (including metformin) (%)	NR	–	18.0	–
Sulfonylurea use (%)	NR	–	68.6	–
Antihypertensive medication (%)	64.9	NR	29.6	12.3
Lipid-lowering medication (%)	40.1	NR	1.2	0.4

^a^Unadjusted excess mortality was calculated as the difference in percentage mortality between control and diabetes groups

NR, not reported

**Fig. 1:**
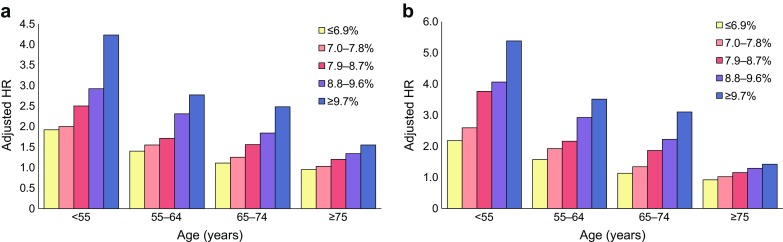
Death from (**a**) any cause and (**b**) from a cardiovascular cause in participants with type 2 diabetes vs control participants. Data shows findings from the Swedish National Diabetes Register [9]. Mean HbA_1c_ levels are indicated as follows: yellow bars, <51.9 mmol/mol (≤6.9%); pink bars, 53.0–61.5 mmol/mol (7.0–7.8%); red bars, 60.7–71.6 mmol/mol (7.9–8.7%); purple bars, 72.7–81.4 mmol/mol (8.8–9.6%); blue bars, >82.5 mmol/mol (≥9.7%). Overall, 77,117 of 435,369 participants with type 2 diabetes (17.7%) died from any cause, as compared with 306,097 of 2,117,483 control participants (14.5%) (adjusted HR 1.15 [95% CI 1.14, 1.16]). *p* values for the interaction term between time-updated mean HbA_1c_ or renal disease status and time-updated age categories were <0.001 in all models. This figure is available as a downloadable slide

Figure 1 shows the adjusted HRs for all-cause mortality (Fig. 1a) and cardiovascular death (Fig. 1b) in individuals with diabetes in the Swedish study vs control participants, in relation to HbA_1c_ level and age [9]. All-cause mortality and cardiovascular death were related to HbA_1c_ in all age groups but were much more pronounced in younger people (<55 years of age). In those older than 75 years, the adjusted HR for all-cause mortality and cardiovascular death was only 1.5 and 1.4, respectively, in those with an HbA_1c_ ≥ 82.5 mmol/mol (≥9.7%), and 1.20 and 1.15, respectively, in those with an HbA_1c_ of 62.8–71.6 mmol/mol (7.9–8.7%). Remarkably, participants between 65 and 74 years of age without albuminuria and with an HbA_1c_ ≤ 51.9 mmol/mol (≤6.9%) had a lower risk in terms of all-cause mortality compared with control participants; the risk was also lower among participants ≥75 years with an HbA_1c_ ≤ 61.7 mmol/mol (≤7.8%) than among control participants.

## Overtreatment and undertreatment of older patients with type 2 diabetes

In older adults with diabetes and multiple serious comorbidities and functional limitations, the harm of intensive glycaemic control likely exceeds the benefits. Among people ≥65 years old, glucose-lowering agents with a risk of hypoglycaemia (insulin and sulfonylureas) were the second most common medications associated with emergency department visits or hospitalisations [30] reported to the US Food and Drug Administration (FDA). The frequent overtreatment of older patients with diabetes, or those with complex comorbidities, has been well documented. Many studies showed that older patients and/or patients with complex or poor health were under tight glycaemic control (often aiming for HbA_1c_ < 53.0 mmol/mol [<7.0%]) and a large proportion received medication associated with hypoglycaemia (sulfonylureas or insulin) [31–33]. This is especially problematic in older patients with dementia who are at much greater risk of hypoglycaemia compared with those without dementia [34] and may be at an added risk of drug interactions due to polypharmacy [35].

In patients with high clinical complexity, intensive treatment significantly increased the risk-adjusted probability of severe hypoglycaemia [36] from 1.74% with standard treatment to 3.04% with intensive treatment. Given the heterogeneity of older patients with type 2 diabetes, an individualised approach is warranted to avoid overtreatment of frail older individuals and undertreatment of those who are otherwise healthy, as recently observed in a large Canadian observational study [33]. In this study, more than half of those with high clinical complexity had HbA_1c_ levels ≤53 mmol/mol (≤7.0%), whereas, in those with HbA_1c_ levels ≥53 mmol/mol (≥7.0%) and low clinical complexity, there was often no up-titration or initiation of additional antihyperglycaemic agents [33].

### High risk of severe hypoglycaemia in older people with type 2 diabetes and comorbidities

Hypoglycaemia is the key rate-limiting step for optimising glycaemic control. It is more common in older individuals with diabetes because of impaired renal and hepatic metabolism with slower counterregulatory mechanisms, polypharmacy or non-adherence to medications, as well as erratic or poor food intake. Since older people with type 2 diabetes exhibit higher complexity, in part due to the higher rate of comorbidities, individualisation of glycaemic targets and treatment strategies is warranted to avoid harm and maximise benefits [37–39]. The recently published trends from 2006 to 2013 [40] in drug use, glycaemic control and rates of severe hypoglycaemia in large cohorts of individuals with diabetes in the USA provide clinically relevant information. Out of the 700,000 individuals analysed in 2013, 50.8% were older than 65 years and 22% were older than 75 years. Rates of severe hypoglycaemia were significantly higher among the oldest compared with the younger individuals (events per 100 person-years: >75 years, 2.3; 65–74 years, 1.3; <65 years, 0.9), and rates of severe hypoglycaemia were particularly high among individuals with two or more comorbidities (3.5 events per 100 person-years) compared with those with no comorbidities (0.4 events per 100 person-years).

There is increasing epidemiological evidence that severe hypoglycaemia may be associated with an increased risk of CVD among people with type 2 diabetes. In a recent very large retrospective cohort study of 58,000 Japanese individuals with diabetes [41], severe hypoglycaemia was strongly associated with the risk of CVD (multivariate-adjusted HR 3.39). Older age (HR 1.24 [95% CI 1.02, 1.52]), long duration of diabetes (HR 1.58 [95% CI 1.14, 2.20]) and a higher Charlson Comorbidity Index (HR 1.14 [95% CI 1.05, 1.23]) were predictors for the development of severe hypoglycaemia, but the strongest predictor was the use of insulin (HR 7.05 [95% CI 4.68, 10.60]). A study using continuous glucose monitoring in older individuals (>69 years) with poor glycaemic control (HbA_1c_ > 63.9 mmol/mol [>8.0%]) found an unexpectedly high frequency of hypoglycaemic episodes (<2.8 mmol/l [50 mg/dl]) in almost half [42]. Based on their findings, the authors concluded that simply relaxing HbA_1c_ goals may not be sufficient to protect frail older adults against hypoglycaemia. Especially in the light of an association between hypoglycaemia and CVD, many older people with diabetes who use insulin and/or sulfonylureas may benefit from a switch to antihyperglycaemic regimens including drugs that do not induce hypoglycaemia, such as metformin, dipeptidyl peptidase-4 (DPP-4) inhibitors, sodium–glucose cotransporter-2 (SGLT-2) inhibitors, pioglitazone or glucagon-like peptide-1 (GLP-1) receptor agonists.

## Renal impairment is common in older people with type 2 diabetes

CKD is a frequent finding in older people with type 2 diabetes [43] and needs to be detected and followed up for selection of the appropriate glucose-lowering drug and dose adaptation when progression of renal impairment occurs [44, 45]. In a retrospective analysis of a large cohort (*n* = 71,092, enrolled in the Kaiser Permanente Northern California Diabetes Registry) of older people with type 2 diabetes, CKD stage 3 (eGFR 30–59 ml min^−1^ [1.73 m]^−2^) was found in 32% and CKD stage 4 (eGFR 15–30 ml min^−1^ [1.73 m]^−2^) in 2.6% of participants [43]. Owing to the decline in cardiovascular death in older people in recent years, more of those with type 2 diabetes and CKD, in particular those presenting with macroalbuminuria, will be at high risk of developing end-stage renal disease (ESRD). In the Action in Diabetes and Vascular Disease: Preterax and Diamicron Modified Release Controlled Evaluation (ADVANCE) study, intensive glucose control with insulin and gliclazide [46] significantly reduced the risk of ESRD by 65% (the number needed to treat was only 41), but increased the risk of severe hypoglycaemia, which was associated with an increase in cardiovascular death [47].

Clemens et al [48] recently reported on the prescriptions of glucose-lowering medication in a large population-based study (Ontario, Canada) of 144,252 older adults (mean age, 78 years) with diabetes and CKD, including individuals on dialysis. Although there were trends towards prescription of safer antihyperglycaemic medication in patients with CKD between 2004 and 2013, up to 49% of those with CKD stages 3–5 and those receiving chronic dialysis were prescribed glibenclamide (known as glyburide in the USA and Canada), and up to 28% with CKD stages 4–5 and those receiving chronic dialysis were prescribed metformin. Although the use of metformin has a beneficial effect in patients with type 2 diabetes and moderate CKD [49], it should not be prescribed in those with severe renal impairment, since a dose-dependent mortality was observed when metformin was used in individuals with CKD stage 5 [50]. In a recent population-based study [51] of >120,000 individuals with diabetes who were new users of a non-insulin glucose-lowering agent, individuals with an eGFR <30 ml min^−1^ [1.73 m]^−2^ using only sulfonylureas had a fivefold increased risk of hypoglycaemia compared with users of metformin only (HR 4.96 [95% CI 3.76, 6.55]). As a result of the very high risk of hypoglycaemia, sulfonylureas should not be used in older patients with impaired renal function. In particular, glibenclamide should be completely avoided, since the associated risk of hypoglycaemia was found to be extremely high (HR 7.48). Individuals with diabetes now have an improved life expectancy but with this comes a longer duration of the disease and its complications, which translates into a greater need for the use of multiple safe agents to maintain glycaemic control. Currently, safer glucose-dependent glycaemic control with DPP-4 inhibitors may be the best choice of treatment in those with diabetes with impaired kidney function [52].

## Treatment targets in older patients with diabetes

There is substantial uncertainty about optimal glycaemic control in older adults with type 2 diabetes mellitus. Until 2010, guidelines recommended HbA_1c_ targets below 53 mmol/mol (7.0%) or 47.5 mmol/mol (6.5%) without any reference to specific glucose-lowering treatments, patient age, diabetes duration or pre-existing CVD [53]. Studies evaluating the effect of intensive glucose lowering (Action to Control Cardiovascular Risk in Diabetes [ACCORD], ADVANCE and Veterans Affairs Diabetes Trial [VADT]) failed to demonstrate a reduction in all-cause mortality and cardiovascular mortality [54–56]. The high frequency of severe hypoglycaemia in these studies was related to the percentage of participants using insulin in the intervention arms [57] and was associated with higher mortality rates [47, 58]. Based on the results of these three landmark studies, Ismail-Beigi et al [38] and the ADA/EASD consensus group [39] proposed individualising glycaemic targets in type 2 diabetes according to the presence or absence of vascular complications, patient age and duration of disease.

In recent years, several international expert groups (the European Diabetes Working Party for Older People, the ADA, the International Diabetes Federation [IDF] and Diabetes Canada (formerly the Canadian Diabetes Association) have provided consensus statements and detailed guidance on the management of diabetes in older people [35, 59–62]. Most of the recommendations are relatively similar; the targets for HbA_1c_, BP and LDL-cholesterol levels according to functional status in older people with type 2 diabetes as proposed by the ADA, IDF and Diabetes Canada are summarised in Table 2.

**Table 2:** Individualised targets for HbA_1c_, BP and LDL-cholesterol in older adults according to health status, proposed by the ADA, IDF and Diabetes Canada

Target	ADA		IDF		Diabetes Canada	
	Patient group	Target	Patient group	Target	Patient group	Target
HbA_1c_,mmol/mol (%)	Healthy	<58.5 (7.5)	Functional/independent	53.0–58.5 (7.0–7.5)	Healthy	≤53.0 (7.0)
	Complex/intermediate	<63.9 (8.0)	Functional/dependent	53.0–63.9 (7.0–8.0)		
	Very complex/poor health	<69.4 (8.5)	Frail	<69.4 (8.5)	Frail	≤69.4 (8.5)
BP,mmHg	Healthy	<140/90	Functional/independent	<140/90		<130/80
	Complex/intermediate	<140/90				
	Very complex/poor health	<150/90	Frail	<150/90		
LDL-cholesterol, mmol/l (mg/dl)		Statins unless contraindicated		<2.07 (<80)		≤2.07 (≤80)

Recent results from the National Health and Nutrition Examination Surveys (NHANES) suggest that poorly controlled diabetes is associated with worse outcomes, including in older people. After a follow-up of 8.9 years, an HbA_1c_ >63.9 mmol/mol (>8.0%) was associated with an increased risk of all-cause and cause-specific mortality in older adults with diabetes [63]. In a UK population-based cohort study [64], >25,000 individuals with type 2 diabetes in the age range of 80 to 89 years were followed-up for a median of 2 years; 35% had a previous diagnosis of CHD, while 11% had previously suffered a stroke. A U-shaped relationship between HbA_1c_ and mortality was observed, with the lowest mortality in people with a baseline HbA_1c_ of 53 mmol/mol (7.0%) to 57 mmol/mol (7.4%), whereas mortality in those with low (<42 mmol/mol [<6.0%]) or high (≥69 mmol/mol [≥8.5%]) HbA_1c_ values was significantly higher.

A multifactorial intervention strategy aiming to reduce all cardiovascular risk factors is now generally recommended to reduce the burden of macrovascular and microvascular complications in people with type 2 diabetes. BP targets are still controversial, in particular concerning protection against stroke and progression of kidney disease. Recently, the ADA and EASD changed their recommended BP targets from 130/80 mmHg to 140/90 mmHg [65]. According to their recommendations, moderate BP control in older people with diabetes according to health status is an appropriate strategy for reducing vascular risk without causing harm. The ADA and EASD guidelines also recommend a target LDL-cholesterol of 2.59 mmol/l (100 mg/dl) for primary prevention and 1.81 mmol/l (70 mg/dl) for secondary prevention in high-risk individuals. For individuals with diabetes who are >75 years of age, there are limited data regarding the benefits and risks of statin therapy. In older patients with a limited life expectancy, any benefits of statins could be counterbalanced by the risks associated with age, polypharmacy, frailty and severe myalgia. Statin therapy should be individualised based on risk profile; for example, high-intensity statin therapy, if well tolerated, is still appropriate and recommended for older adults with manifest atherosclerotic vascular disease [66].

## Individualisation of glucose-lowering therapy

### Metformin mono- and combined therapy

Metformin is the leading glucose-lowering drug worldwide and is also the first-line oral medication for hyperglycaemia in older adults [67]. The rise, fall and revival of metformin in the therapy of type 2 diabetes is unique, changing its position from a devil in the 1970s to an angel in the last 20 years [68]. Metformin has been in clinical use for diabetes treatment for 60 years and all potential advantages and disadvantages are very well known [69]. The glucose-lowering effect is profound [70] and relatively long-lasting. Because metformin’s mechanism of action predominantly involves a reduction in hepatic glucose production, it rarely causes hypoglycaemia when used alone [69]. Many properties, such as low costs, weight loss of 2–3 kg [70, 71], antiatherogenic effects, as well as the option to be combined with all other drugs, explain why metformin is recommended in all guidelines as a first-line therapy. According to a recent large UK observational study [64], metformin was used in more than 60% of patients with diabetes aged between 80 and 89 years. Some older people may experience intolerable gastrointestinal discomfort and decreased appetite with metformin use. Further, as aforementioned, kidney function declines with age and some caution is needed since metformin is contraindicated in those with renal failure. The FDA advises against the use of metformin in patients with an eGFR of <30 ml min^−1^ [1.73 m]^−2^.

In a very large prospective study [71], the durability of the glucose-lowering effects of rosiglitazone, metformin or glibenclamide monotherapy was analysed in 4360 patients with a short duration of diabetes (<2 years in 97% of the study group). The cumulative incidence of treatment failure of metformin (defined as fasting plasma glucose >10.0 mmol/l [180 mg/dl]) after 5 years was 21% and, after 4 years, only 36% of those using metformin had an HbA_1c_ < 53 mmol/mol (<7.0%). In principle, all six classes of glucose-lowering drugs proposed by the ADA–EASD consensus statement [67] could be considered as second-line treatment in addition to metformin but, as stated above, in older patients with high comorbidity, drugs inducing hypoglycaemia may not be the best choice. Nonetheless, owing to the economic burden, sulfonylureas are still widely used, even in extremely elderly patients with complex type 2 diabetes. A relatively better choice, when the economic situation does not allow for the use of modern drugs, may be gliclazide, a sulfonylurea with the lowest risk of hypoglycaemia that may be safer than other compounds [72, 73].

### DPP-4 inhibitors, SGLT-2 inhibitors and GLP-1 receptor agonists

Experience with the use of newer glucose-lowering drugs (DPP-4 inhibitors, SGLT-2 inhibitors, GLP-1 receptor agonists) is increasing. Importantly, these drugs are not associated with an increased risk of hypoglycaemia [67]. DPP-4 inhibitors have been examined in several prospective RCTs in older patients with type 2 diabetes, and their efficacy, safety and very low risk of hypoglycaemia compared with sulfonylureas or placebo has been documented [74–76]. In the UK, since 2009, a significant trend towards fewer hospitalisations for hypoglycaemia was recently reported among older adults (65–80 years) with type 2 diabetes, which may be explained by the decreasing use of sulfonylureas [77]. Several large studies of DPP-4 inhibitors (Saxagliptin Assessment of Vascular Outcomes Recorded in Patients with Diabetes Mellitus [SAVOR], Examination of Cardiovascular Outcomes with Alogliptin versus Standard of Care [EXAMINE], Trial Evaluating Cardiovascular Outcomes with Sitagliptin [TECOS]) have not shown any cardiovascular benefit but have demonstrated safety [78–81]. However, some caution against the use of DPP-4 inhibitors in patients with heart failure is needed, in particular when renal impairment is present [82].

The baseline characteristics of participants in all 11 cardiovascular outcome trials in those with diabetes published up to now (October 2017) are summarised in electronic supplementary material (ESM) Table 1. Five out of the 11 trials investigated newer glucose-lowering drugs (pioglitazone, empagliflozin, liraglutide, semaglutide, canagliflozin) and demonstrated a significant reduction in three-point major adverse cardiovascular events (MACE; cardiovascular death, non-fatal myocardial infarction and non-fatal stroke), whereas six drugs (insulin glargine, saxagliptin, alogliptin, sitagliptin, lixisenatide and exenatide) showed safety but no change in cardiovascular mortality and morbidity when compared with placebo [78–80, 83–89]. Since the mean age of the participants at baseline was about 60–65 years (ESM Table 1) and about half were older than 65 years, the positive findings of these cardiovascular outcome trials appear to be relevant for drug selection in older patients with established CVD. Both empagliflozin [85] and liraglutide [87] were associated with an impressive reduction in cardiovascular death and all-cause mortality, which led to a change in the labelling of the drugs by the FDA and the European Medicines Agency (EMA). The number needed to treat to prevent one cardiovascular death was only 46 and 77 for empagliflozin and liraglutide, respectively, over 3 years. Hospitalisation owing to heart failure was only reduced by SGLT-2 inhibitors, which is relevant with regard to the high prevalence of heart failure in older people with type 2 diabetes. Interestingly, a decline in renal events was found with empagliflozin, canagliflozin and liraglutide when used in patients with long-standing type 2 diabetes and a history of CVD. Important renal events were decreased by all three drugs; however, a significant reduction of doubling of serum creatinine was only achieved by empagliflozin [85]. Overall mortality, hospitalisation owing to heart failure and incidence or worsening of nephropathy were also significantly reduced by empagliflozin vs placebo in patients aged 65–75 years and >75 years at baseline [90–92]. Since participants in the cardiovascular outcome trials were selected based on the presence of established CVD, it is unknown whether SGLT-2 inhibitors and GLP-1 receptor agonists may also have beneficial effects on the heart and kidney in very early stages of the disease.

Subgroup analyses of different age groups (<60 years and ≥60 years) from cardiovascular outcome trials with significant effects on the primary outcome (MACE, myocardial infarction or stroke) are summarised in Table 3. Interestingly, among older participants, substantial effects on three-point MACE were seen with empagliflozin, canagliflozin and exenatide, while liraglutide and semaglutide did not appear to confer any additional benefit. This effect observed with empagliflozin may be owing to the beneficial effect of this drug on heart failure, which is more common in older age [93].

**Table 3:** Results of subgroup analyses of cardiovascular outcome trials with significant effects in primary outcome

Trial	Glucose-lowering agent	Primary outcome	Age group	*n*	Percentage of participants with event		HR (95% CI)	*p* value for interaction
					Tested glucose-lowering agent	Placebo		
EMPA-REG [85]	Empagliflozin	Three-point MACE	All ages	7020	10.5	12.1	0.86 (0.74, 0.99)	
			<65 years	3893	9.7	9.3	1.04 (0.84, 1.29)	
			≥65 years	3127	11.4	15.5	0.71 (0.59, 0.87)	0.01
LEADER [87]	Liraglutide	Three -point MACE	All ages	9340	13.0	14.9	0.87 (0.78, 0.97)	
			<60 years	2321	11.7	14.8	0.78 (0.62, 0.97)	
			≥60 years	7019	13.5	14.9	0.90 (0.79, 1.02)	0.27
IRIS [94]	Pioglitazone	MI or stroke	All ages	3876	9.0	11.8	0.76 (0.62, 0.93)	
			<65 years	2168	7.5	10.2	0.73 (0.55, 0.97)	
			≥65 years	1708	10.9	13.8	0.79 (0.60, 1.03)	0.72
CANVAS [89]	Canagliflozin	Three -point MACE	All ages	10,142	NR	NR	0.86 (0.75, 0.97)	
			<65 years		NR	NR	0.91 (0.76, 1.10)	
			≥65 years		NR	NR	0.80 (0.62, 0.97)	0.26
SUSTAIN [86]	Semaglutide	Three -point MACE	All ages	3297	6.6	8.9	0.74 (0.58, 0.95)	
			<65 years	1699	6.2	8.3	0.74 (0.52, 1.05)	
			≥65 years	1598	6.9	9.4	0.72 (0.51, 1.02)	0.95
EXSCEL [81]	Exenatide	Three -point MACE	All ages	14,752	11.4	12.2	0.91 (0.83, 1.00)	
			<65 years	8813	9.4	8.9	1.05 (0.92, 1.21)	
			≥65 years	5939	14.4	17.2	0.80 (0.71, 0.91)	0.005

CANVAS, Canagliflozin Cardiovascular Assessment Study; EMPA-REG, Cardiovascular Outcome Event Trial in Type 2 Diabetes Mellitus; EXSCEL, Exenatide Study of Cardiovascular Event Lowering; IRIS, Insulin Resistance Intervention after Stroke; LEADER, Liraglutide Effect and Action in Diabetes: Evaluation of Cardiovascular Outcome Results; MI, myocardial infarction; NR, not reported; SUSTAIN, Trial to Evaluate Cardiovascular and Other Long-Term Outcomes with Semaglutide in Subjects with Type 2 Diabetes

### Thiazolidinediones

The use of thiazolidinediones, including pioglitazone, has declined drastically in recent years (from 28.5% in 2016 to 5.6% in 2013 in a population of 1.66 million patients with type 2 diabetes in the USA [40]), because of safety issues (increase in heart failure and bone fractures) and the availability of newer glucose-lowering drugs. Pioglitazone may be an option in carefully selected patients with diabetes [94] with previous stroke or transient ischaemic attack (TIA); in the Insulin Resistance Intervention after Stroke (IRIS) study, participants without diabetes but with insulin resistance and previous stroke or TIA showed a significant reduction in a subsequent stroke by 24% [95] and acute coronary syndrome by 29% [96] when pioglitazone was added to other cardiovascular-protective drugs. These data are in line with the significant reduction in repeat stroke by 48% in the Prospective Pioglitazone Clinical Trial in Macrovascular Events (PROactive), when individuals with diabetes and previous stroke were randomised to pioglitazone instead of placebo [97].

### Glucose-lowering treatment in frail patients

Very little is known about the effect and risk of glucose-lowering treatment in frail patients with type 2 diabetes. Recently, two different glucose-lowering treatment strategies were compared in vulnerable (moderately ill and/or frail) patients aged ≥65 years with type 2 diabetes whose individual HbA_1c_ targets were not met with diet/exercise and/or oral antihyperglycaemic medications [98]. Both the glucose-dependent strategy (a GLP-1 receptor agonist plus a non-sulfonylurea oral glucose-lowering drug) and the glucose-independent strategy (insulin glargine plus a sulfonylurea) reduced HbA_1c_ similarly, by 12.0 mmol/mol (1.1%) from baseline to 55.2 mmol/mol (7.2%). Incidences of total, documented symptomatic, and asymptomatic hypoglycaemic events were significantly lower in patients treated with the glucose-dependent strategy vs the glucose-independent strategy (10.2% vs 53.8%, 5.1% vs 36.6%, 8.2% vs 32.3%, respectively; *p* < 0.001 for each), indicating that a glucose-dependent strategy is preferable in the treatment of frail patients with diabetes.

### Adverse effects

Considerations for the selection of glucose-lowering agents in the treatment of older people with type 2 diabetes are summarised in Table 4. Detailed information is given for each drug class, including risk for hypoglycaemia, advantages, disadvantages, results in cardiovascular outcome trials, dose adaptation in renal impairment and costs. In terms of adverse effects, gastrointestinal side effects are not uncommon with the use of metformin and GLP-1 receptor agonists and a very small risk of pancreatitis may exist for the use of DPP-4 inhibitors. Meanwhile, bone fractures have been reported for pioglitazone and canagliflozin, and mycotic genital infections are seen in about 5–10% of patients (more so in women than men) using SGLT-2 inhibitors. Recently, a small increased risk of foot amputation was also reported for canagliflozin [89]. Euglycaemic diabetic ketoacidosis may occur when older people with severe insulin deficiency receive SGLT-2 inhibitors, or when it is triggered, for example, by reductions in insulin dosage, low energy and fluid intake, intercurrent illness or alcohol. Remarkably, the well-monitored participants in the Empagliflozin Cardiovascular Outcome Event Trial in Type 2 Diabetes Mellitus (EMPA-REG Outcome) [85] and Canagliflozin Cardiovascular Assessment Study (CANVAS) [89] did not show any increase in diabetic ketoacidosis following intervention. Unfortunately, the relatively high costs of modern glucose-lowering drugs are an important limitation for their use in countries with a limited economy.
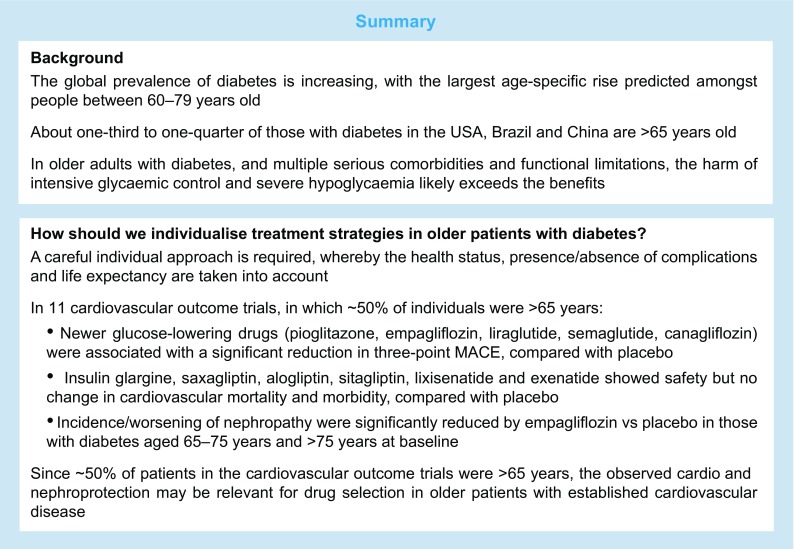

**Table 4:** Considerations for selection of glucose-lowering drugs in older patients with type 2 diabetes

Drug	Risk of hypoglycaemia	Advantages	Disadvantages	Results in CVOTs	Dose adaptation in renal impairment	Cost
Metformin	Low	In clinical use for 60 years	GI side effects (nausea, diarrhoea) Use cautiously when eGFR <45 ml min^−1^ [1.73 m]^−2^ Contraindicated when GFR <30 ml min^−1^ [1.73 m]^−2^ Lactic acidosis (very rare)	NR	Yes	Very low
Sulfonylurea (glibenclamide, glipizide, gliclazide, glimepiride)	High (highest for glibenclamide, lowest for gliclazide)		Weight gain Cardiovascular safety controversial	NR	Yes	Very low
Pioglitazone	Low	Antiatherogenic effects	Water retention Heart failure Bone fractures	Beneficial effect on CV risk	Yes	Low
DPP-4 inhibitor (sitagliptin, alogliptin, saxagliptin, linagliptin)	Low	Glucose-dependent effect Well tolerated	Increased risk of heart failure hospitalisation (saxagliptin) Small increased risk of pancreatitis	Neutral effects on CV risk (sitagliptin, alogliptin, saxagliptin)	Yes (exception: linagliptin)	High
GLP-1 receptor agonist (exenatide, lixisenatide, liraglutide, dulaglutide)	Low		GI side effects (nausea, vomiting, anorexia) Subcutaneous administration	Beneficial effect on CV risk for liraglutide Neutral effects for lixisenatide and exenatide	With caution	Very high
SGLT-2 inhibitor (canagliflozin, empagliflozin, dapagliflozin)	Low	Beta cell-independent effect Can be combined with all other classes of glucose-lowering drugs	Dehydration Genitourinary infections Case reports of euglycaemic ketoacidosis Bone fractures and foot (toe) amputation (canagliflozin)	Beneficial effects on CV risk for empagliflozin and canagliflozin	Yes Contraindicated when eGFR <45 ml min^−1^ [1.73 m]^−2^	High
Insulin/insulin analogues	Very high	High efficacy	Problematic in older patients with complex disease, renal impairment or dementia	Neutral effect on CV risk	Yes	High (very high with SMBG)

CVOT, cardiovascular outcome trial; GI, gastrointestinal; NR, not reported; SMBG, self-monitoring of blood glucose

## Future research

In the next 30 years, older adults are predicted to make up the majority of those with type 2 diabetes. Thus, extensive research efforts are required, in particular for patients with a long life expectancy. A diabetes control and complications trial for older adults with diabetes stratified into participants with and without CVD would be very helpful to establish targets for HbA_1c_, BP and LDL-cholesterol levels for primary and secondary prevention of complications. Future research should aim to answer the many questions that remain in this field, such as when do some drugs induce more harm than benefit in vulnerable patients with complex disease? Further, it would be interesting to investigate whether newer glucose-lowering drugs (which recently demonstrated significant cardiovascular and renal benefit in those with a history of CVD) are also superior to the widely used more established drugs (e.g. metformin and sulfonylureas) in older individuals, or whether their benefit is only applicable to the prevention of severe hypoglycaemia in this age group, which is observed in about 1% of patients using sulfonylureas.

## Conclusion

In summary, a carefully designed individual approach is needed for the treatment of older patients with type 2 diabetes, in which the health status, presence or absence of complications, and life expectancy should be taken into account. The heterogeneity of older people with diabetes must be considered. There is a need to individualise all therapeutic strategies based on specific factors that predict benefits and risks, including the functional and cognitive status of patients and the burden of comorbidities. Glucose-dependent drugs that do not induce hypoglycaemia are preferable because older patients with impaired kidney function are especially vulnerable to this adverse event. Since, in general, older people with diabetes but without complications now survive much longer than in our recent history, a multifactorial intervention to lower HbA_1c_ (to 53 mmol/mol [7.0%]), BP (to <140/90 mmHg) and lipids (i.e. use of statins) may be helpful to reduce the high risk of future development of heart failure, stroke, myocardial infarction and renal impairment. In 2017, the focus of the treatment of patients with type 2 diabetes is expanding to include the prevention of cardiovascular morbidity and mortality by use of specific glucose-lowering drugs with documented benefits in cardiovascular outcome trials. Empagliflozin and liraglutide have the best evidence to date among glucose-lowering agents for the reduction of cardiovascular death in patients with manifest CVD and type 2 diabetes. SGLT-2 inhibitors may be the preferred drug class in individuals with type 2 diabetes presenting with heart failure [99], which is much more common than previously believed [100]. Finally, early glycaemic control, avoidance of hypoglycaemia, and multifactorial cardiovascular risk factor-targeted interventions remain the cornerstones of cardiovascular and renal protection, even in older people with type 2 diabetes.

### Contribution statement

Both authors drafted and critically revised this article. Both authors approved the final version.

## Electronic supplementary material

ESM Downloadable slide (PPTX 105 kb)

ESM Table 1 (PDF 46.4 kb)

## Abbreviations

CKD: Chronic kidney disease
CVD: Cardiovascular disease
DPP-4: Dipeptidyl peptidase-4
ESRD: End-stage renal disease
FDA: Food and Drug Administration
GLP-1: Glucagon-like peptide-1
IDF: International Diabetes Federation
MACE: Major adverse cardiovascular event
SGLT-2: Sodium–glucose cotransporter-2
TIA: Transient ischaemic attack
