# Supplementary material for: Diabetes in the older patient: heterogeneity requires individualisation of therapeutic strategies
Source: Diabetologia. 2018 Feb 7;61(7):1503–16. doi: 10.1007/s00125-018-4547-9 (PMC6445482; doi:10.1007/s00125-018-4547-9)
Supplement: Supplementary file 1 — (PPTX 105 kb) [file 125_2018_4547_MOESM1_ESM.pptx]

## Slide 1
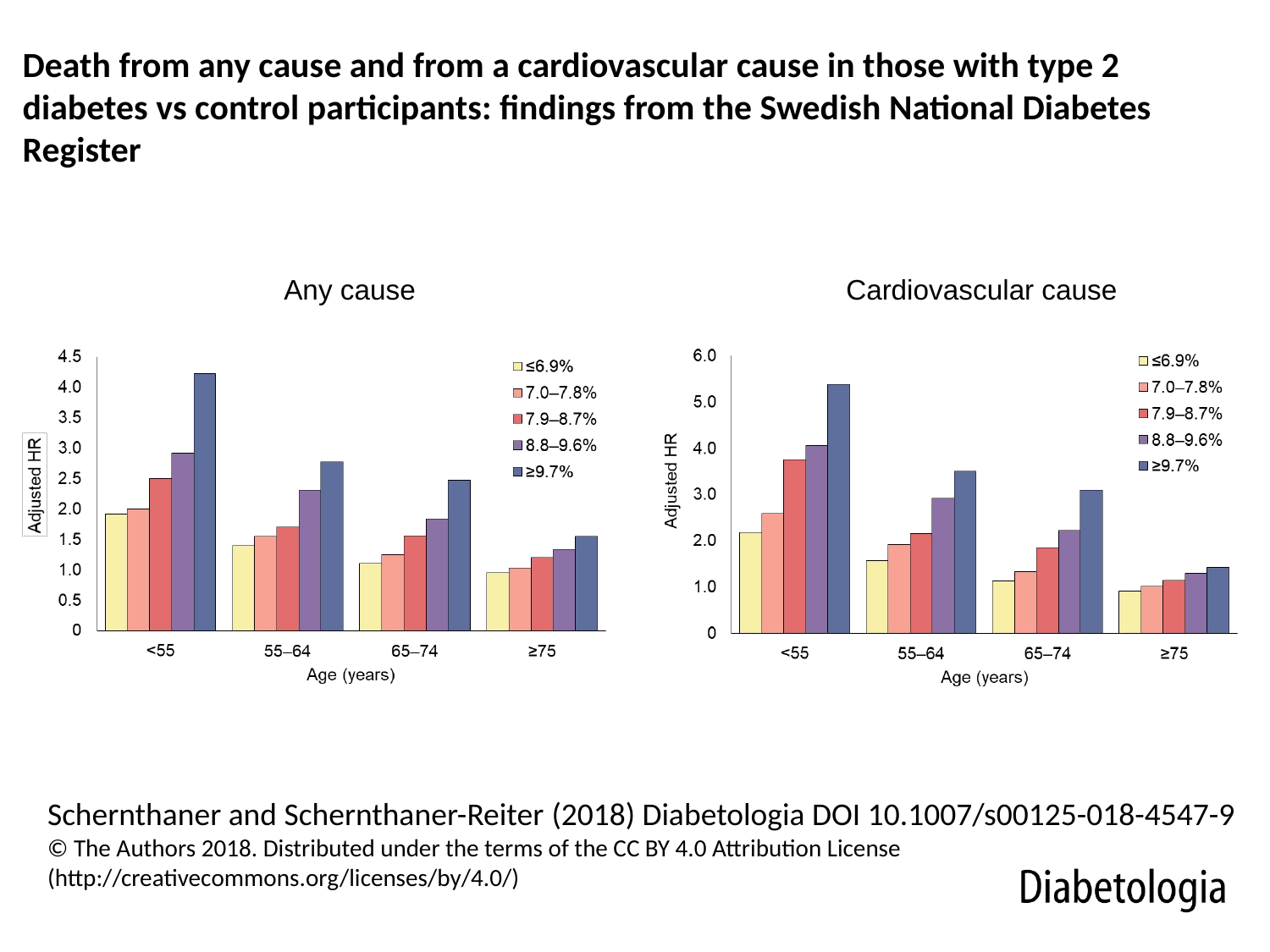

Death from any cause and from a cardiovascular cause in those with type 2 diabetes vs control participants: findings from the Swedish National Diabetes Register
Any cause
Cardiovascular cause
Schernthaner and Schernthaner-Reiter (2018) Diabetologia DOI 10.1007/s00125-018-4547-9
© The Authors 2018. Distributed under the terms of the CC BY 4.0 Attribution License (http://creativecommons.org/licenses/by/4.0/)
