# Supplementary material for: Diabetes in the older patient: heterogeneity requires individualisation of therapeutic strategies
Source: Diabetologia. 2018 Feb 7;61(7):1503–16. doi: 10.1007/s00125-018-4547-9 (PMC6445482; doi:10.1007/s00125-018-4547-9)
Supplement: Supplementary file 2 — (PDF 46.4 kb) [file 125_2018_4547_MOESM2_ESM.pdf]

ESM Table 1 Baseline characteristics of participants in cardiovascular outcome studies

	SGLT-2 inhibitors		GLP-1 agonists				DPP-4 inhibitors		
Variable	EMPA-REG [85]	CANVAS (pooled) [89]	ELIXA [88]	LEADER [87]	SUSTAIN [86]	EXSCEL [81]	SAVOR [78]	EXAMINE [79]	TECOS [80]
Baseline	Empagliflozin	Canagliflozin	Lixisenatide	Liraglutide	Semaglutide	Exenatide	Saxagliptin	Alogliptin	Sitagliptin
Age (years)	63.1 (63.1±8.6 for empagliflozin; 63.2±8.8 for placebo)	63.3 (±8.3)	60.3 (59.9±9.7 for lixisenatide; 60.6±9.6 for placebo)	64.3 (±7.2)	64.6 (±7.4)	62.0 (±6.0)	65.1 (65.1±8.5 for saxagliptin; 65.0±8.6 for placebo)	61.0	65.5 (±8.0)
Duration of diabetes (years)	12	13.5 (±7.8)	9.3 (9.2±8.2 for lixisenatide, 9.4±8.3 for placebo)	12.9 (12.8±8.0 for liraglutide, 12.9±8.1 for placebo)	13.9 (±8.1)	12.0	10.3 (IQR 5.2–16.7 for saxagliptin; 5.3–16.6 for placebo)	7.2 (7.1 for alogliptin; 7.3 for placebo)	11.6 (±8.1)
BMI (kg/m^2)	30.6	32.0	30.2	32.5	32.8	32	31	29	30
Insulin (%)	48	50	39	45	58	46	41	30	23
HbA_{1c} (mmol/mol)	65.0	66.1	60.7	71.6	71.6	63.9	63.9	63.9	55.2
HbA_{1c} (%)	8.1	8.2	7.7	8.7	8.7	8.0	8.0	8.0	7.2
Prior CVD(%)	99	66	100	~81	~83	73	79	100	100
Types of prior CVD	MI, CHD, CVD, PVD	MI, CHD, CVD, PVD	ACS <180 days	≥50 years + CHD/CVD/PVD or CKD or ≥60 years + ≥1 CV risk factor	≥50 years + CHD/CVD/PVD or CKD or ≥60 years + ≥1 CV risk factor	CHD, CVD, PVD	≥40 years + CHD/CVD/PVD or ≥55/60 years + risk factors	ACS <90 days	CHD, CVD, PVD
Hypertension (%)	95	90.0	76.4	92	92.8	90	82	83	86
Follow-up (years)	3.1	3.6	2.1	3.8	2.1	3.2	2.1	1.5	3.0

ACS, acute coronary syndrome; CANVAS, Canagliflozin Cardiovascular Assessment Study; CV, cardiovascular; CVD, cerebrovascular disease; ELIXA, Evaluation of Lixisenatide in Acute Coronary Syndrome; EMPA-REG, Cardiovascular Outcome Event Trial in Type 2 Diabetes Mellitus; EXSCEL, Exenatide Study of Cardiovascular Event Lowering; IQR, interquartile range; LEADER, Liraglutide Effect and Action in Diabetes: Evaluation of Cardiovascular Outcome Results; MI, myocardial infarction; NR, not reported; PVD, peripheral vascular disease; SAVOR, Saxagliptin Assessment of Vascular Outcomes Recorded in Patients with Diabetes Mellitus; SUSTAIN, Trial to Evaluate Cardiovascular and Other Long-Term Outcomes with Semagultide in Subjects with Type 2 Diabetes; TECOS, Trial Evaluating Cardiovascular Outcomes with Sitagliptin. Values provided are means ± SD unless otherwise stated.
